# Physical exercise and its associated factors among Ethiopian pregnant women: a cross-sectional study based on the theory of planned behavior

**DOI:** 10.1186/s40359-022-00847-z

**Published:** 2022-06-09

**Authors:** Abebaw Addis, Wallelign Alemnew, Ayenew Kassie, Simegnew Handebo

**Affiliations:** 1 Amhara Regional State Health Bureau, Bahir Dar, Ethiopia; 2 Department of Health Education and Behavioural Sciences, College of Medicine and Health Sciences, University of Gondar, Gondar, Ethiopia; 3 School of Public Health, Saint Paul’s Hospital Millennium Medical College, Addis Ababa, Ethiopia

**Keywords:** Intention, Physical exercise, Theory of planned behavior, Pregnant women

## Abstract

**Introduction:**

Women in Ethiopia prefer sedentary behavior and are physically inactive during pregnancy; this increases the risks of pregnancy-related complications. Therefore, this study aimed to assess physical exercise and its associated factors among pregnant women attending Antenatal Care at Debermarkose Referral Hospital, Northwest Ethiopia: using the theory of planned behavior.

**Methods:**

An institution-based cross-sectional study was conducted from 23rd February to 10th June, 2020. A systematic random sampling technique was used to select 333 study participants. Data were collected with face-to-face interviews using a pretested structured questionnaire. Epi-info and STATA version 14.0 were used for data entry and analysis respectively. Multivariable linear regression analysis was done to identify the association between dependent and independent factors, and *p* < 0.05 was used as a cutoff to determine statistical significance at multivariable logistics regressions.

**Result:**

A total of 333 pregnant women were participated in this study with response rate of 98%. The proportion of variance (*R*^2^) in intention accounted for the factors was 0.79. The magnitude of intention to do physical exercise during the current pregnancy was 3.8 ± 2. primary level of education (β = 0.43; 95% CI 0.25, 0.61), 2nd trimester gestational age (β = 0.19; 95% CI 0.04, 0.33), 3rd trimester gestational age (β = 0.17; 95% CI 0.02, 0.32), Attitude (β = 0.34; 95% CI 0.19, 0.49), subjective norm (β = 0.12; 95% CI 0.01, 0.23) and perceived behavioral control (β = 0.45; 95% CI 0.33, 0.57) were significantly associated with pregnant womens’ intention to do physical exercise during pregnancy period.

**Conclusion and recommendations:**

This study provided that the theory of planned behavior could be successfully applied to identify the factors related to the intention to do physical exercise during pregnancy The program designers who work on pregnancy better consider providing Information education and communication to change the attitude, work on influencing significant individuals and barriers and enabling factors.

## Introduction

Physical exercise during pregnancy is associated with short and long-term benefits for the mother and fetus [1, 2]. Healthy pregnant women should follow the American College of obstetrician and gynecologists (ACOG) and society of obstetrician and gynecologist of Canada (SOGC) recommendations that states healthy pregnant women should be physically active throughout pregnancy by achieving at least 150 min of moderate intensity aerobic physical exercise per week to reduce the risk and acquire benefit from the recommended exercise [3, 4]. The risks are pregnancy-induced hypertension, gestational diabetes mellitus, prolonged duration of labor, low back pain, delivering macrosomia baby, influences on the mode of delivery, and excessive weight gain [5]. Studies indicated that aerobic exercise reduces the risk of gestational diabetes millets by 55% throughout the pregnancy and 25% during early pregnancy exercise [6–9]. Furthermore, aerobic physical exercises during pregnancy shorten the duration of first stage labor and reduce Cesarean by 15% [10–12].

Ethiopia is one of the Sub-Saharan Africa countries that has high maternal mortality ratio (MMR) reported at 412 [13]. Even though the country has in good progress in the reduction of maternal mortality rate still it has a wide gap as compared to developed countries which are 12 [14]. In the reduction of maternal complication during pregnancy physical exercise has great importance as the study confirmed [15]. A study done in Eastern Ethiopia showed that 16.3% of maternal death during pregnancy is due to hypertensive disorder of pregnancy [16]. Another study done in Ethiopian urban areas on level and intensity of physical activity showed that pregnant mothers acquired 1.46 MET (Metabolic Equivalent) which is considered as physically inactive and low as compared to women from low income countries [17]. Recent studies also show that novelty-seeking and personality traits are associated with higher level of physical activity [18, 19].

According to the theory of planned behavior three kinds of considerations: beliefs about the likely consequences of the behavior (behavioral beliefs), beliefs about the normative expectations of others (normative beliefs), and beliefs about the presence of factors that may facilitate or impede performance of the behavior (control beliefs) guide human behavior [20]. In combination with those indirect constructs, direct attitude toward the behavior, direct subjective norm, and direct perception of behavioral control lead to the formation of a behavioral intention [21]. It has been applied to studies of the relations among beliefs, attitudes, behavioral intentions and behaviors in various fields [20, 21]. Studies have also applied the constructs of theory of planned behavior model to predict intention to do physical exercise during pregnancy, hence attitude, perceived behavioral control and subjective norm had direct relation with intention to do physical exercise [22–24]. However, to date, no research has applied the theory of planned behavior model to study the magnitude of pregnant mothers’ intention to do physical exercise in Ethiopia. Furthermore, the factors that increase or decrease intentions to do physical exercise are not well identified in the study area. Therefore, this study aims to assess the magnitude of intention to do physical exercise and its associated factor among pregnant women who were attending antenatal care at Debermarkose referral hospital based on the theory of planned behavior.

## Methods

### Study design and setting

An institution-based cross-sectional study was conducted from 23rd February to 10th June 2020 among Debermarkose referral hospital of antenatal care attendant pregnant women. It is situated in the Amhara National Regional state at 376 km away from Addis Ababa city in North West direction of the country.

### Sampling and sample size determination

As far as the knowledge of the investigators there was no study conducted in Ethiopia on intention to do physical activity among pregnant women. Therefore, a pilot study was conducted to get the standard deviation and mean intention. The sample size was determined using the single population mean and double population mean formula as below;

$$\begin{aligned} {\text{n}} & = \left( {{\text{Z}}\upalpha /_{2} } \right)^{2} {\varvec{\delta}}^{2} \\ {\text{d}}^{2} & = 1.96^{2} \times 5^{2} = 323 \\ & \quad (0.04 \times 13.64)^{2} \\ \end{aligned}$$

where n = sample size, Zα/_2_ = 95%confidence level, δ = standard deviation (5), d = degree of accuracy desired to set (0.04) and, mean intention (13.64). By adding a 5% non-response rate the final sample size was 339 participants. Those participants with placenta Previa, premature rupture of membrane, and pregnancy-induced hypertension were excluded.

To select a sample of 339 antenatal care attendants from the total 819 attendants (total number of ANC attendants in the Hospital), we assigned a consecutive number from 1 to N in the ANC registration book. Then sampling started by selecting an element from the list at random and then every kth element in the frame was selected, where k was the sampling interval.

### Measurement

*Intention* Intention to do physical exercise is the outcome variable, it is the mother’s readiness to do physical exercise during the current pregnancy. It was measured with four items having a five-point Likert scale (Cronbach’s α = 0.96). The questions were asked like “I intend to do physical exercise during the pregnancy period.” The responses were 1 “strongly disagree”, 2 “disagree”, 3 “neutral”, 4 “agree”, and 5 “strongly agree”. The composite score ranged from four to twenty and the high score indicate high intention [25].

*Regular physical exercise* is an aerobic physical exercise that lasts 30 min continuously at least 3 days a week like brisk walking, stationary cycling, and swimming [26].

*Direct attitude* Individual feelings and beliefs about physical exercise. It was measured with seven items having a five- Likert scale (Cronbach’s α = 0.89). The questions were asked, “For me exercising regularly for 30 min per day at least 3 days a week during my pregnancy will be…” The response was 1 “bad” …0.5 “good”. The composite score ranges from seven to thirty five the high score indicates a high attitude towards physical exercise [27].

*Direct subjective norm* Perception of individuals of the social pressure to do physical exercise. It was measured with five items having five-point Likert scale (Cronbach’s α = 0.95). Subjective norm questions were asked, “Most people who are important to me think that I should exercise regularly during my pregnancy period”. The response was 1 “strongly disagree”, 2 “disagree”, 3 “neutral”, 4 “agree” and 5 “strongly agree”. The composite score ranges from five to twenty five the higher score indicates high social influence towards physical exercise [25].

*Perceived behavioral control* The perceived ability of an individual to control factors that influenced physical exercise. It was measured with five items having a five-point likert scale (Cronbach’s α = 0.91). The questions were asked, “For me doing regular physical exercise during my pregnancy is…” The responses were 1 “very difficult” …0.5 “easy”. The composite score ranges from five to twenty five and the higher score indicates higher perceived ability of individuals to control factors [21, 25].

*Previous experience* Individuals who have done exercise during the antenatal period at least once in life time. And the questions were asked like “have you ever done physical exercise during pregnancy period?”.

*Indirect Subjective norm* It is composed of normative belief i.e. one’s belief about perceived social pressure to do physical exercise and motivation to comply. The normative questions were asked as “my husband thinks that I should do physical exercise during pregnancy.” The responses were (1) “strongly disagree”, (2) “disagree”, (3) “neutral”, (4) “agree”, (5) “strongly agree”. And the motivations to comply questions were asked like “how much do you care about what your husband think you should do physical exercise during pregnancy?” The responses were (1) “not at all”, (2) “not”, (3) “neutral”, (4) “somewhat” and (5) “very much”. It was assessed by a total twelve items for normative belief measuring and six motivations to comply with measuring items. The final value of the variable was produced by computing the sum product of normative belief and motivation to comply [21]. The internal consistency of items was (Cronbach’s α = 0.92).

*Indirect Perceived behavioral control* It is composed of control belief i.e. one’s perception to do physical exercise and perceived power. The questions of control belief were asked like “how the access of the facilities may affect your physical exercise during pregnancy. The responses were (1) “not at all”, (2) “not”, (3) “neutral”, (4) “somewhat” and (5) “very much”. And questions of perceived power were asked like “if I feel that I don’t have access of the facilities, it would be more difficult to do physical exercise. The questions were asked like “I intend to do physical exercise during the pregnancy period.” The responses were (1) “strongly disagree”, (2) “disagree”, (3) “neutral”, (4) “agree”, (5) “strongly agree”. It was assessed by a total of ten items for perceived control and five for perceived power measuring items. The final value of the variable was produced by computing the sum product of perceived control and perceived power [21]. The internal consistency of items was (Cronbach’s α = 0.84).

*Knowledge* knowledge about physical exercise during pregnancy was asked with eleven items. The items were developed from works of various literatures [28–31] and have Cronbach’s α = 0.76 for the internal consistency of the items. Each response was valued with “1” for correct responses and “0” for incorrect responses. The knowledge score of each individual was obtained by summing up all the ten items and the expected score ranged from 0 to 11.

### Data collection procedure and data quality control

The data were collected using a structured, pretested and interview administered questionnaire prepared by the investigators after reviewing different literatures [14, 21, 25, 32, 33]. Elicitation study was conducted using semi-structured interview to explore relevant salient behavioral belief among the study population towards physical exercise, normative belief that influence them to do or not to do physical exercise and about control beliefs. Sixteen pregnant women participated in the elicitation study. Salient beliefs were identified and the commonly held beliefs were used in the development of the questionnaire for the main study. The questionnaire was initially prepared in English and then translated to Amharic (local language) then back to English. Four data collectors and one supervisor were recruited from health professionals holding BSc degree. Content validity test was conducted by participating 6 experts from midwifery, health behavior and Obstetrics and gynecologists. It was determined by Item-level Content Validity Index (I-CVI) of 0.78 or higher, Scale level Content Validity Index by Universal Agreement (S-CVI/UA) 0f 0.8 or higher and Scale level Content Validity Index by Average (S-CVI/Ave) 0.9 or higher.

### Data processing and analysis

Prior to the use of the instrument the reliability analysis or internal consistency test for the TPB constructs carried out and the result was above 0.7 which is acceptable (Cronbach’s alpha); it shows how closely related a set of items are as a group. All collected data was coded, cleaned and entered in to epi-data version 4.6 and was transferred to SPSS version 20 statistical software for its analysis. Descriptive analysis was used to see frequency distribution, mean and standard deviation. Correlation analysis was done between indirect and direct TPB variables in order to see the relation between them. Linear regression analysis was fitted to test the association between dependent variable and independent variables. The assumptions of normality of dependent variable which is intention statistically using skewness and kurtosis test i.e., Z value of skewness and it was found to be between − 1.96 and + 1.96 so that it was normally distributed. Linearity assumption was checked using scatter plot of the standardized residuals versus the predicted values from the regression analysis. Multicollinearity assumptions were tested by variance inflation factor (VIF) and the value of all variables were below ten. The assumption of outlier was tested using box plot and there was no outlier. R^2^ was used for the ability of explanatory variables to explain dependent variables. First all independent variables were entered in simple linear regression and those variables whose *p* value less than 0.2 were included in multiple linear regressions. Unstandardized β coefficient was used to interpret the effect of factors associated to intention to do physical exercise. Variables with *p* value less than 0.05 at 95%confidence interval were considered as statistically significant.

## Results

A total of 333 participants were engaged with a response rate of 98.2%. Regarding to the religion, most (73.3%) were orthodox Christian followers. About one-fourth (24%) of the participants had no formal education, and the majority (90.7%) were resided in urban while 60.7% were house wife in occupation. Almost all (99.1%) of the women were married (Table 1).

**Table 1:** Sociodemographic characteristic of pregnant women in Debermarkose referral hospital, North West, Ethiopia 2020 (n = 333)

Variable	Category	Frequency (n)	Percentage (%)
Age	< 25	63	18.92
	25–35	222	66.67
	35 and above	48	14.41
Marital status	Married	330	99.1
	Divorced	1	0.3
	Widowed	2	0.6
Level of education	No formal education	25	7.5
	Primary level	84	25.2
	Secondary level	128	38.4
	College and above	41	12.3
Husbands level of education	No formal education	80	24.02
	Primary level	42	12.7
	Secondary level	99	29.9
	College and above	112	33.8
Residence	Urban	302	90.7
	Rural	31	9.3
	Orthodox Christian	244	73.3
Religion	Muslim	47	14.1
	Protestant	42	12.6
Occupation of women	House wife	202	60.7
	Daily laborer	40	12
	Merchant	57	17.1
	Government employee	34	10.2
Husband’s occupation	Daily laborer	95	28.5
	Merchant	84	25.2
	Farmer	45	13.5
	Government employee	107	32.1
Monthly family income	≤ 2000	131	39.3
	2001–5000	130	39.0
	≥ 5001	72	21.6

### Physical activity characteristics of pregnant women

About 6.1% had physical activity with the majority (95.2%) of participants did brisk walking and the remaining engaged in jogging. Nearly half (47.6%) reported they engage for 15 min and while most (81%) them engaged in physical activity irregularly per week while about 14.3% did twice a week.

### Knowledge towards about antenatal exercise

The mean knowledge score of the respondents was 5.28 (SD ± 2.38). The majority (81%) of the participants knew that aerobic exercise like brisk walking during pregnancy prevent excessive weight gain and 90% reduce risk of high blood pressure, and more than half (54%) knew that it is not recommended for a woman with vaginal bleeding (Table 2).

**Table 2:** Knowledge of pregnant mothers towards aerobic exercise (brisk walking for 30 min per day at least 3 days in a week during pregnancy) in Debermarkose referral hospital, North West, Ethiopia 2020

	Answer (yes)	Frequency	Percent (%)
Reduces back pain	Yes	94	28.2
Prevents excessive weight gain	Yes	270	81
Reduces risk of high blood pressure	Yes	304	91.2
Reduces risk of gestational diabetes	Yes	205	61.5
Reduces the likely hood of cesarean section delivery	Yes	59	17.7
Helps more rapid recovery after delivery	Yes	59	17.7
Aerobic exercise (brisk walking) is not recommended for woman with-------?			
Vaginal bleeding during pregnancy	Yes	180	54
Severe anemia during pregnancy	Yes	264	79.2
Uncontrolled type 1 DM during pregnancy	Yes	37	11.1
Uncontrolled hypertension during pregnancy	Yes	29	8.7
Severe head ache	Yes	260	78

### Obstetric characteristics of women

The majority of the participants 144 (43.2%) were multigravida, 101 (30.3%) were multiparous and 88 (26.4%) were primigravida. Most of the respondents were belongs with in third trimester pregnancy 114 (34.2%).

### Theory of planned variables

The median score of direct attitude, subjective norm and perceived behavioral control was 3.57, 4 and 3.6 respectively. Indirect measures of theory of planned behavior were assessed and the median score of indirect attitude was 104 ± 83. The median intention was 3.8 ± 2.

### Correlation of theory of planned behavior variables with intention

All of the theory of planned behaviour variables had significantly correlated with intention at *p* value of < 0.05. Indirect attitude had highest correlation followed by direct perceived behavioural control. Indirect perceived behavioural control had negative correlation with intention (Table 3).

**Table 3:** Correlation of theory of planned behavior variables with intention

	I	DATT	DSN	DPBC	IDATT	ISN	IPBC
I							
DATT	0.75*	1					
DSN	0.76*	0.81*	1				
DPBC	0.78*	0.83*	0.85*	1			
IDATT	0.79*	0.78*	0.78*	0.82*	1		
ISN	0.67*	0.75*	0.83*	0.79*	0.71*	1	
IPBC	− 0.16*	− 0.27*	− 0.21*	− 0.28*	− 0.23*	− 0.19*	1

*I* intention, *IDATT* indirect attitude, *DATT* direct attitude, *ISN* indirect subjective norm, *DSN* direct subjective norm, *IPBC* indirect perceived behavioral control, *DPBC* direct perceived behavioral control

* Significant at a *p*-value less than 0.05

### Multiple linear regressions

The level of education, occupation, experience of regular exercise, gestational age, knowledge about physical exercise and all the direct theory of planned behaviour variables were with a *p* value of < 0.2 in simple linear regression.

In multiple linear regression, variables which increased intention to do physical exercise were identified and these were level of education, gestational age, attitude, subjective norm, and perceived behavioural control were significantly associated variables with intention to do regular physical exercise with at 95% confidence interval.

The variance explained by intention to do regular physical exercise from factors was 79%. The standardized regression coefficient suggested that direct perceived behavioral control (β = 0.52) was the strongest factor of intention to do regular physical exercise followed by direct attitude (β = 0.27) and direct subjective norm (β = 0.11).

The participant level of education was significantly and positively associated with intention to do regular physical exercise. Participants who attained primary level education (1–8) were 0.43 (B = 0.43:95% CI 0.25–0.61) times more likely to increase intention than those with no formal education provided that other variables are constant. Moreover, the intention of mothers in second and third trimester increased by 0.19 (B = 0.19; 95% CI 0.04–0.33) and 0.17 (B = 0.17; 95% CI 0.02–0.32) than those mothers in first trimester.

For a score increase of direct attitude, intention to do regular physical exercise increased by 0.34 (B = 0.34; 95% CI 0.19–0.49). A positive score increase in direct perceived behavioral control could result in increase of intention to do physical activity by 0.45 (B = 0.45; 95% CI 0.33–0.57). For a positive unit increase in a subjective norm could result increase of intention to do regular physical exercise by 0.12 (B = 0.12; 95% CI 0.01–0.023) (Table 4).

**Table 4:** Multiple linear regression of intention to do regular physical exercise and associated factor among pregnant mothers in Debermarkose referral hospital, 2020

Variable	β	Β	*p* value	95% CI for	B
*Education level*					
No formal education (ref)					
Primary level	0.19	0.43	< 0.01*	0.25	0.61
Secondary level	0.09	0.21	0.05	− 0.01	0.41
College and above	0.06	0.18	0.35	− 0.20	0.58
*Residence*					
Urban (ref)					
Rural	0.03	0.16	0.17	− 0.06	0 .38
*Participants occupation*					
House wife (ref)					
Daily laborer	− 0.05	− 0.05	0.08	− 1.46	0.08
Merchant	− 0.05	− 0.05	0.16	− 1.19	0.19
Government employee	0.01	0.01	0.92	− 1.42	1.59
*Experience of exercise*					
No (ref)					
Yes	− 0.06	− 0.05	0.718	− 0.34	0.23
*Gestational age*					
First trimester (ref)					
Second trimester	0.08	0.19	0.01*	0.04	0.33
Third trimester	0.17	0.17	0.02*	0.02	0.32
*Gravidae*					
One (ref)					
Two to four	0.12	0.26	0.32	− 0.26	0.78
Above four	0.17	0.38	0.17	− 0.17	0.94
*Parity*					
None (ref)					
One	− 0.12	− 0.39	0.15	− 0.94	0.15
Two and above	− 0.16	− 0.34	0.21	− 0.87	0.19
*Knowledge*	0.02	0.1	0.35	− 0.02	0.04
*TPB variables*					
Attitude	0.27	0.34	< 0.01*	0.19	0.49
subjective norm	0.11	0.12	0.03*	0.01	0.23
perceived behavioral control	0.52	0.45	< 0.01*	0.33	0.57

* Significant at a *p*-value less than 0.05

## Discussion

This study assessed intention to do physical exercise and associated factors among pregnant women attending ANC at Debermarkose referral hospital using the theory of planned behavior. The participants’ level of education, gestational age and theory of planned behavior variables such as perceived behavioral control, attitude and subjective norm were associated variables of intention to do physical exercise among pregnant women. The current study revealed that the mean value of intention to do regular physical exercise was 3.8. It suggested that the average intention to do physical exercise was almost agreeing. This finding is supported by other study [34].

This study showed that participants’ level of education is an important associated factor of intention to do exercise regularly. This finding is consistent with studies conducted in the Tigray region of Ethiopia and India [35]. It might be due to that those individuals with higher level of education have better health literacy regarding the benefits of exercise during pregnancy, and knew how to do and what types of exercises are recommended. Therefore, they might plan to do exercise to gain the above benefits and avoid the risks [36, 37].

Our study found that gestational age is a significant associated factor of intention to do physical exercise. Participants in second and third trimesters had increased intention than those in first trimester. It is supported by a study conducted in Brazil [38]. Most pregnancy related complications such as gestational hypertension, Gestational DM and fear of labor complications occurred in the second and third trimesters so that pregnant women may engage in physical exercise to prevent those complications [37, 39]. Furthermore, pregnant mothers gain weight in these trimesters and health professionals could recommend them to do physical activity during antenatal visits [40]. Therefore, program planners who work on pregnancy, better to focus on second and third trimesters to increase physical exercise.

This study revealed that perceived behavioral control is an associated factor of intention to do regular physical exercise. This is supported with a study done in Taiwan [41]. This is because of pregnant mothers who are confident, wanted doing regular physical exercises are more intended. This suggests that encouraging pregnant mothers to aim for doing regular physical exercise should involve consideration of doing regular exercise is under their control that means when a pregnant mother able to overcoming barriers to do physical exercise like time constraint, fear of injury, fear of miscarriage may lead to increased intention to do regular exercise. This finding is supported by Ajzen’s theoretical assumption; the more pregnant mothers have a high degree of control over factors that facilitate or imped them made easy to perform what they want [25].

Attitude was a positively associated with intention to do regular physical exercise. The finding is consistent with previous studies conducted on intention to do physical activity [27, 42]. This is because of individuals who believe that regular physical exercises reduce gestational DM, pregnancy induced hypertension and prevent miscarriage are more probable to have favorite feeling towards physical activity and plan to do it [23, 43]. In addition, the finding is supported by the elicitation study done to formulate the indirect attitude. Thus, formation positive attitude of pregnant mothers could increase the physical exercise behavior.

The current study found that subjective norm was a positively associated with intention to do regular physical exercise. This is consistent with a research conducted in Taiwan [41]. This implies that the decision to do physical exercise is made not only by the individuals themselves but also influenced by them significant others such as families, friends, health professionals, spouse and neighbors. As a result interventions to improve doing regular exercise among pregnant women should target those important others as a whole rather than focusing only on pregnant mothers [25].

### Limitations of the study

Since the data was from self-report there could be the probability of the occurrence of recall and social desirability bias. Furthermore, due to the cross-sectional nature of the study, it doesn’t show cause and effect relationship. Recent studies showed that personality trait has an effect on intention to do physical exercise so future researchers better to incorporate personality trait as an explanatory variable.

## Conclusion

Theory of planned behavior could be successfully applied to determine the associated factors of intention to do physical exercise during pregnancy. Factors of intention to do regular physical exercise were direct perceived behavioral control, direct attitude, direct subjective norm, participants level of education, and gestational age. Therefore, information education and communication is required to bring favorable attitude, to reduce perceived berries and interventions should also consider the mother, friend, spouse and health professionals who had influence to her decision.

## Data Availability

The datasets used and/or analyzed during the current study are available from the corresponding author on reasonable request.

## Abbreviations

ACOG: American college of obstetrician and gynaecologists
AOR: Adjusted odds ratio
ATT: Attitude
CI: Confidence interval
DM: Diabetic mellitus
I: Intention
MET: Metabolic equivalent
MMR: Maternal mortality ratio
PBC: Perceived behavioral control
SN: Subjective norms
SOGC: Society of Obstetrician and Gynecologist of Canada
TPB: Theory of planned behaviour
WHO: World Health Organization
